# Situs Ambiguous With Polysplenia With Azygos Continuation of the Inferior Vena Cava (IVC): A Case Report

**DOI:** 10.7759/cureus.107082

**Published:** 2026-04-15

**Authors:** Jad Kabbara, Pasang Sherpa, Enayat Shahidifar, Arman Hemmat

**Affiliations:** 1 Anesthesiology, Lake Erie College of Osteopathic Medicine, Erie, USA; 2 Internal Medicine, Mercy Health - St. Elizabeth Youngstown Hospital, Youngstown, USA

**Keywords:** azygos continuation, congenital venous anomaly, heterotaxy syndrome (hs), incidental radiologic finding, inferior vena cava, polysplenia, situs ambiguous

## Abstract

Situs ambiguous with polysplenia with azygos continuation of the inferior vena cava (IVC) represents an exceptionally rare spectrum of congenital anomalies resulting from the disrupted left-right access development during embryogenesis. Although most reported cases are identified in childhood due to the associated cardiac and biliary malformations, survival into adulthood without major comorbidities is uncommon and rarely documented. We presented the case of a 37-year-old female who presented to the emergency department with left leg swelling and persistent nausea and vomiting. Imaging performed for unrelated symptoms revealed multiple right-sided spleens, a predominantly left-sided liver, and an infrahepatic IVC draining via the azygos system into the superior vena cava (SVC), all of which are findings consistent with sinus ambiguous with polysplenia. This case is unique in demonstrating the incidental asymptomatic presentation of this complex anomaly in an adult without significant cardiobiliary comorbidities. From a radiological standpoint, awareness of this pattern is critical to prevent misinterpretations as simply a vascular obstruction or post-operative change. Clinically, the recognition of this anomaly is essential for surgical planning, central venous access, and interpretation of cross-sectional imaging. Reporting exceptionally rare adult cases like this patient aids in expanding the understanding of this heterotaxy’s variable spectrum and emphasizes the importance of accurate radiological identification in optimizing patient care.

## Introduction

Situs ambiguous with polysplenia and azygos continuation of the inferior vena cava (IVC) describes a rare constellation of congenital anomalies involving the abnormal arrangement of the thoracoabdominal organs and vascular structures. Situs inversus is a term used to describe the complete mirror image reversal of the normal positions of the thoracic and abdominal organs, which differs from the classic situs solitus, which is the normal anatomical position of the organs. The unique combination of both is the development of situs ambiguous, better known as heterotaxy [1]. 

Polysplenia, the presence of multiple small spleens, is an exceptionally rare manifestation of heterotaxy with an estimated prevalence of approximately one in 250,000 live births and is heavily associated with cardiac and biliary abnormalities. The heterotaxy was first described in 1929 and is classically associated with high childhood mortality due to the high prevalence of complex cardiac defects and biliary atresia in affected infants. More than 75% of patients with polysplenia and heterotaxy die before five years of age in historical series [2]. The number of spleens can range from one to 10 or more, with spleens being dispersed either bilaterally or, if presented unilaterally, it is usually tied ipsilaterally to the stomach due to its embryonic origin from the dorsal mesogastrium [3-5]. One of the most consistent findings in heterotaxy associated with polysplenia is the interruption of the hepatic segments of the IVC with continuation through the azygos system, which has been demonstrated on computed tomography (CT) series in up to 74% of patients affected by polysplenia syndrome [3,4].

These abnormalities both stem from the embryological disruption in the left-right axis pattern during early development, which progresses to asymmetric organogenesis and vascular malformation [5]. While most cases of this heterotaxy tend to be detected in infancy due to their severe cardiobiliary defects, this incidental discovery of this anomaly in adulthood is exceedingly rare and carries important clinical implications. Adult cases without significant core morbidities remain under-reported, making every discovery a valuable contribution to understanding this heterotaxis variable spectrum and long-term survivability.

The clinical significance of polysplenia-associated heterotaxy can widely vary from an incidental finding in adults to severe congenital cardiac and biliary malformations associated with high neonatal mortality [5-7]. Cases that present incidentally on imaging without significant cardiac or biliary involvement can reach adulthood asymptomatically. Given the rarity of this constellation of anomalies, we report the incidental findings of a 37-year-old female with situs ambiguous with polysplenia and azygos continuation of the IVC.

## Case presentation

A 37-year-old female presented to the emergency department complaining of right leg pain and swelling with sudden bouts of vomiting that happened suddenly. The patient reported that she woke up with severe right leg pain and does not recall any trauma or hitting her leg prior to the onset of illness. There is associated nausea and vomiting with the leg swelling but no recorded fever or chills. The patient denied the loss of sensation and was able to move both feet and toes; however, there was severe pain reported. Physical examination of the right lower extremity revealed diffuse erythema, warmth, and tenderness extending from the mid-calf to the ankle with significant non-pitting edema. There were no visible skin breaks, puncture wounds, insect bites, or signs of trauma. Distal pulses were intact bilaterally, capillary refill was normal, and sensation and motor function were preserved. Given the acute unilateral lower extremity swelling and pain, deep vein thrombosis was considered in the differential diagnosis. A venous Doppler ultrasound of the right lower extremity was performed and demonstrated no evidence of thrombosis, effectively excluding deep vein thrombosis as the cause of the patient’s symptoms. No fluctuance or crepitus was appreciated. Cardiovascular examination revealed normal heart sounds without murmurs, and no clinical signs of heart failure were noted. Abdominal examination was soft and non-distended without tenderness or palpable organomegaly. No jaundice or scleral icterus was observed. The patient’s past medical history was significant only for obesity and hypertension. She denied any prior history of congenital heart disease, hepatic disorders, chronic pulmonary disease, or prior thromboembolic events. She reported no previous developmental abnormalities and no known family history of congenital or vascular malformations. She had no prior history of recurrent or severe infections suggestive of functional asplenia.

**Figure 1:**
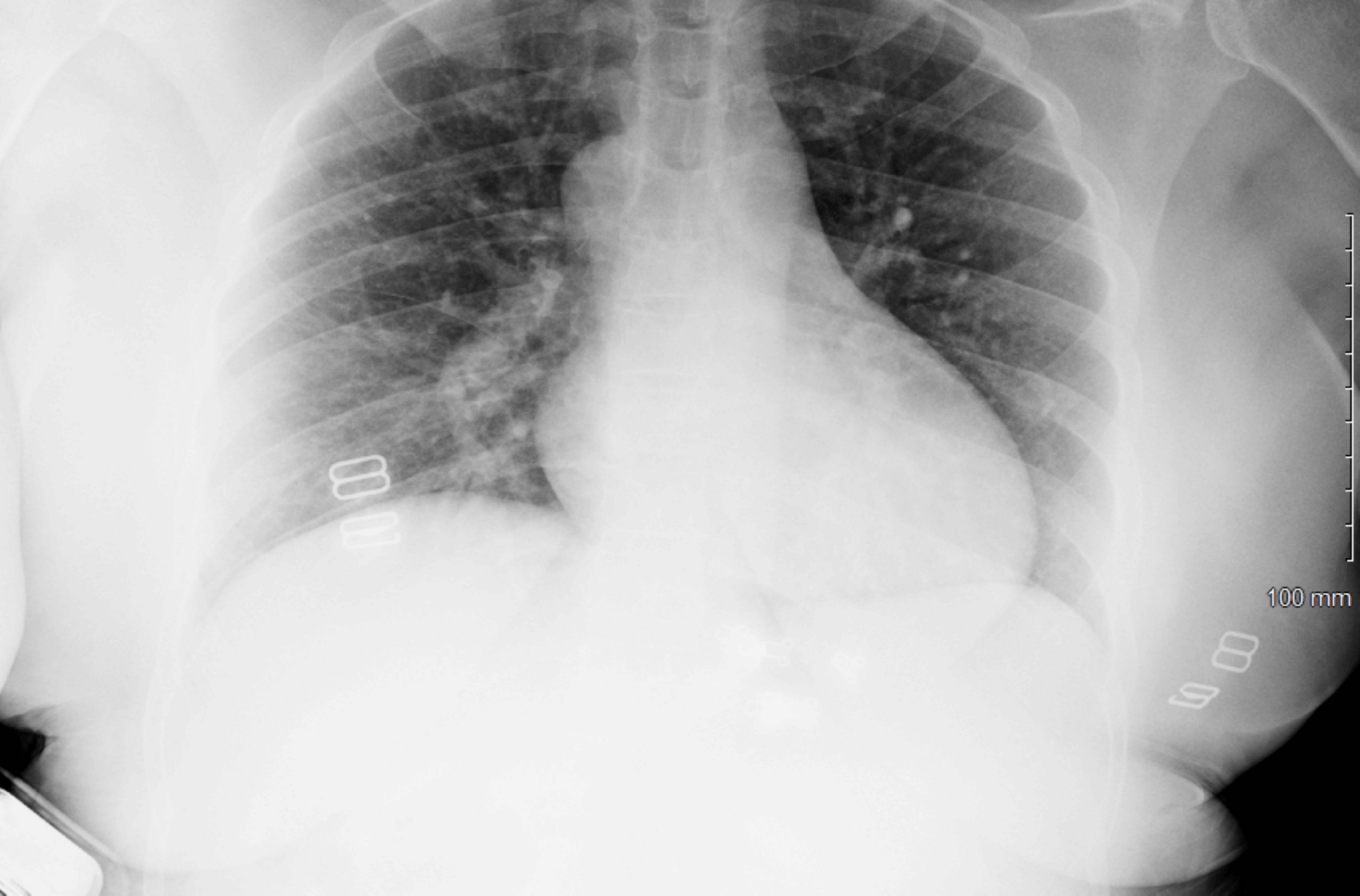
Chest X-ray obtained in the emergency department showing no acute process.

**Figure 2:**
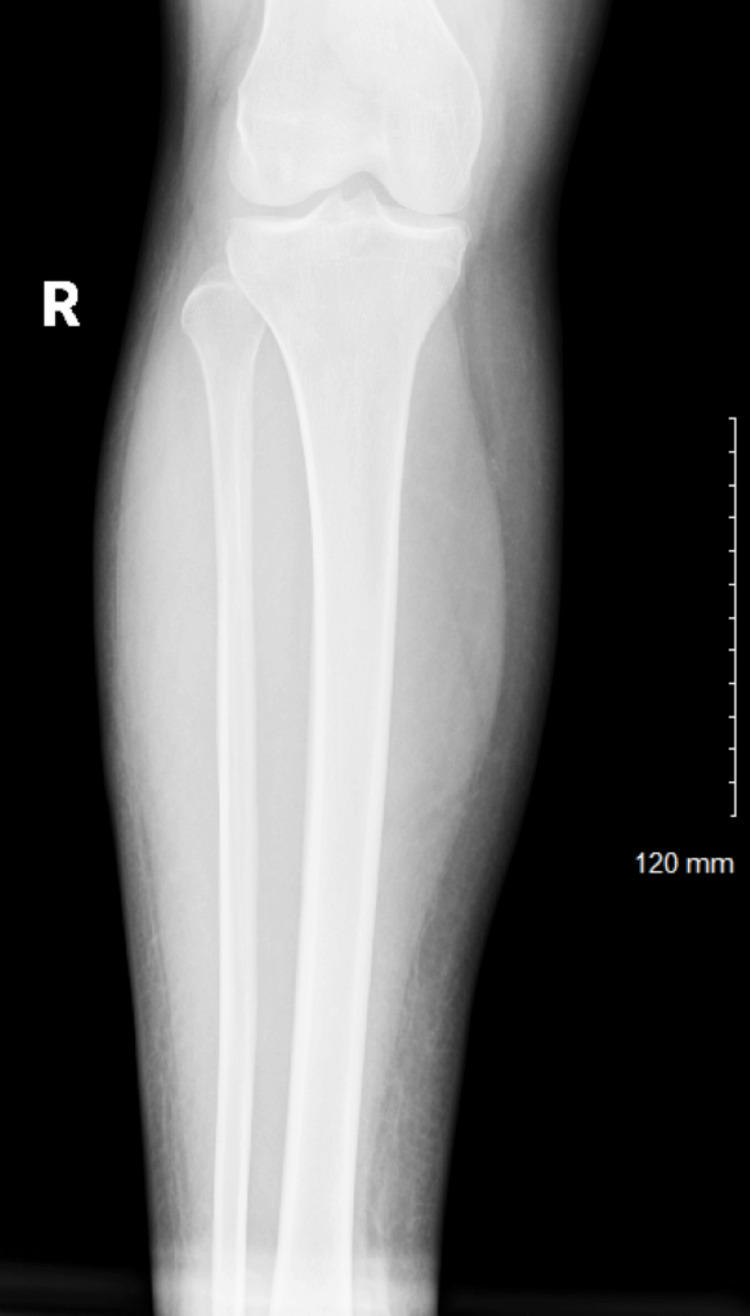
Right tibia fibula showed severe soft tissue edema but no tissue air or osteomyelitis consistent with cellulitis.

**Figure 3:**
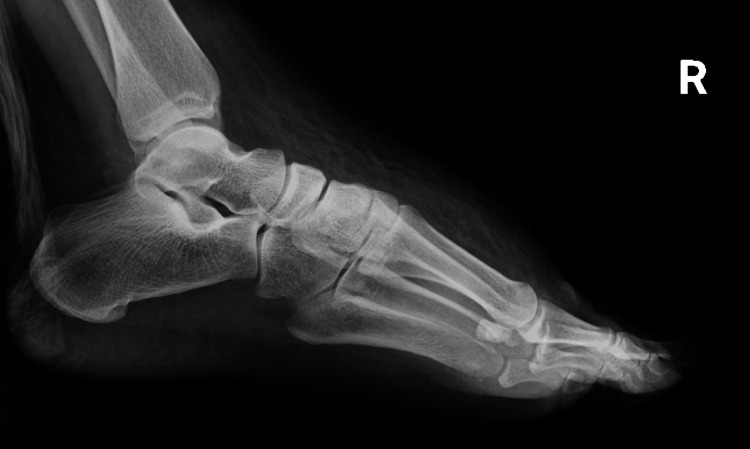
X-ray of the right foot demonstrate severe soft tissue edema without subcutaneous air or osseous changes, consistent with cellulitis.

A chest radiograph was obtained as part of the initial evaluation for nausea, vomiting, and systemic infection to assess for possible pulmonary pathology or cardiomegaly. The study demonstrated a normal cardiac silhouette without dextrocardia and no acute pulmonary process (Figure 1), and an X-ray of the right foot (Figure 2) and right tibia fibula showed severe soft tissue edema without tissue air or radiographic evidence of osteomyelitis. The findings were consistent with cellulitis (Figure 3). Given the sudden onset of nausea and vomiting without an identifiable cause on physical examination and concern for possible intra-abdominal pathology, contrast-enhanced CT of the chest, abdomen, and pelvis was performed for further evaluation. No structural cardiac abnormalities were identified on CT imaging. There was no evidence of atrial or ventricular septal defects, abnormal cardiac positioning, or outflow tract abnormalities. No structural cardiac abnormalities were identified on CT imaging. There was no evidence of atrial or ventricular septal defects, abnormal cardiac positioning, or outflow tract abnormalities. Given the absence of structural cardiac abnormalities on contrast-enhanced CT imaging, a normal cardiac silhouette on chest radiograph, and a lack of clinical signs or symptoms suggestive of cardiac dysfunction, transthoracic echocardiography was not pursued during this admission. In addition, the patient remained hemodynamically stable without murmurs, hypoxia, or features concerning for underlying congenital heart disease, further supporting the decision to defer echocardiographic evaluation. 

**Figure 4:**
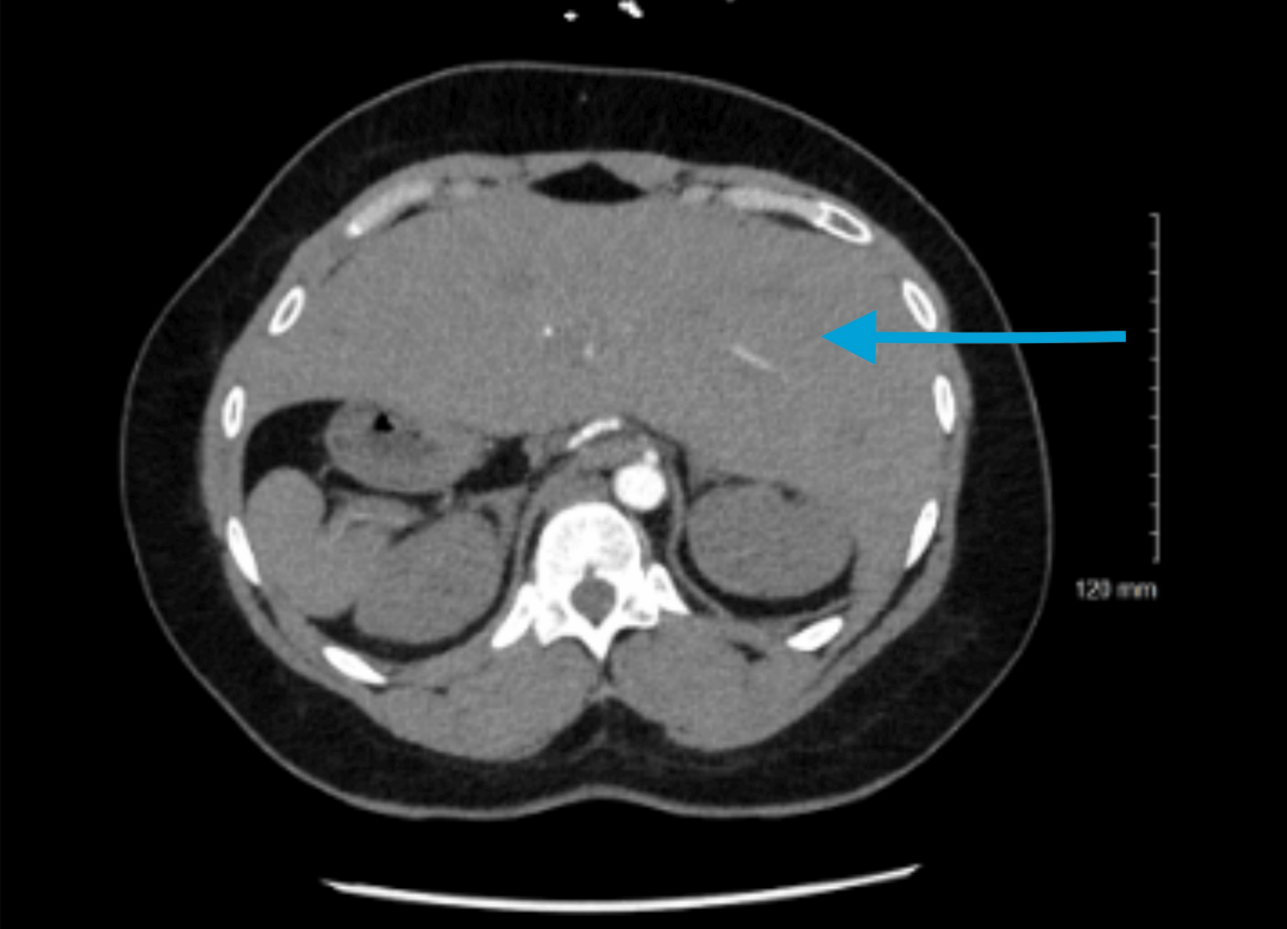
Axial contrast-enhanced CT demonstrates classic features of situs ambiguous with polysplenia. Multiple splenules are visualized along the right upper quadrant, consistent with right-sided polysplenia. The liver is predominantly left-sided, occupying most of the left hemiabdomen.

**Figure 5:**
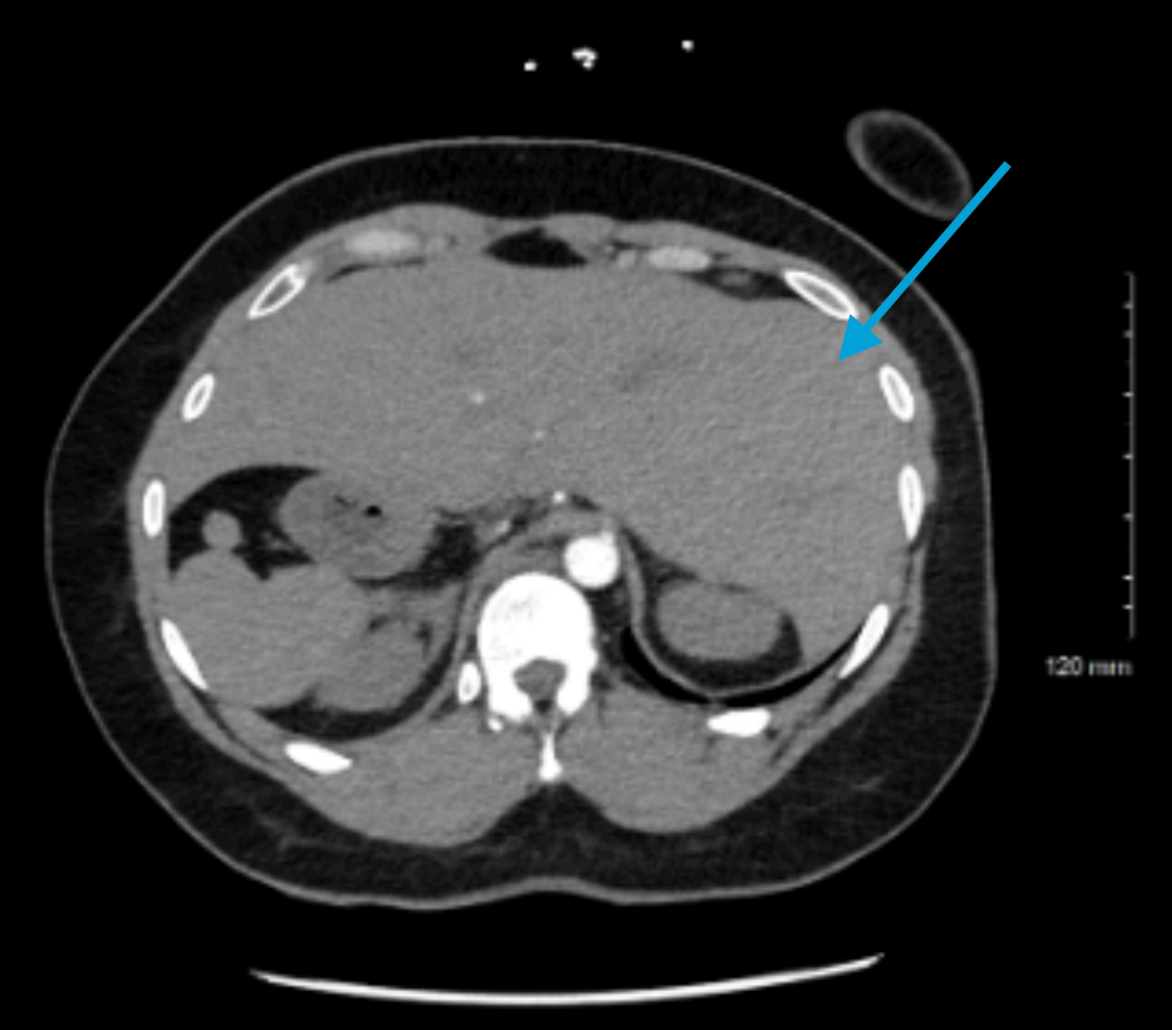
This axial CT image demonstrates features consistent with abdominal heterotaxy most notably multiple splenic nodules clustered within the right upper quadrant reflecting polysplenia. The liver is positioned predominantly on the left producing an atypical visceral arrangement.

The lungs did not demonstrate bilobed symmetry or bronchiectasis. Incidentally, the CT pulmonary with contrast revealed findings that consisted of situs ambiguous with polysplenia and azygos continuation of the IVC, while the vascular course is most clearly appreciated on multiplanar reconstruction, the diagnosis was established based on contrast-enhanced imaging and radiologist interpretation. Multiple spleens were located along the right side of the abdomen in association with a right-sided stomach, while the liver was predominantly positioned on the left side, consistent with situs ambiguous (Figures 4, 5). Five discrete splenules were identified along the right upper quadrant, ranging in size. These findings were confirmed in the official radiology report and reviewed in multiplanar reconstruction. Although the splenules are relatively small and subtle on axial imaging, coronal views clearly demonstrated multiple discrete splenic nodules in the right upper quadrant consistent with polysplenia. No dominant splenic mass was identified in the typical left upper quadrant splenic bed. Further evaluation revealed the infrahepatic IVC drainage superiorly via the azygos vein along the right paravertebral region, leading up to the superior vena cava (SVC), consistent with azygos continuation of the IVC, which is a vascular anomaly frequently associated with polysplenia syndrome. The vascular anomaly was most clearly appreciated on coronal and sagittal multiplanar reconstruction images, which demonstrated interruption of the hepatic segment of the IVC with continuation through a dilated azygos vein ascending along the right paravertebral region into the SVC. The incidental findings did not require immediate intervention, nor did they change the hospital course for this patient; however, it was documented for future surgical and interventional planning. A peripheral smear was not performed during this admission, as there was no clinical suspicion for hematologic abnormality. Given the absence of recurrent infections and the presence of multiple splenic structures on imaging, functional asplenia was considered unlikely. The patient reported receiving routine childhood vaccinations but had not previously received additional vaccinations for encapsulated organisms. Given the incidental nature of the finding and absence of infectious history, referral to immunology was not pursued during this admission, although this was recommended for outpatient follow-up.

The patient was discharged four days after admission with the primary diagnosis of methicillin-resistant *Staphylococcus aureus* (MRSA)-positive cellulitis of the right leg that was actively managed with vancomycin during the hospital stay, which was later transitioned to doxycycline and augmentin upon discharge.

## Discussion

Polysplenia syndrome is a rare heterogeneous condition that is associated with a high mortality rate and infancy, with a majority of the literature on this heterotaxy coming from pediatric cases and surgical series. Reported adult cases of this heterotaxia are exceedingly uncommon and are usually due to incidental findings during imaging for unrelated conditions [8]. Within polysplenia syndrome, the variability that presents in the anatomical structures can be broad, with patients having anomalies varying within the pancreas, such as dorsal pancreatic agenesis leading to a short or even absence of pancreatic tail or intestinal malrotation and pre-duodenal portal veins, in addition to the classic features of multiple spleens and IVC interruptions [7,8]. A previous case report reported a condition of polysplenia with agenesis of the dorsal pancreas and a pre-duodenal portal vein leading to obstructive jaundice in an adult patient [8]. In another case reported by Malki et al., an elderly patient with polysplenia was described, who had situs inversus totalis and an annular pancreas, further illustrating that this heterotaxy can be associated with a wide spectrum of gastrointestinal and vascular anomalies beyond simply the spleen and the heart [9]. Our case adds to this wide spectrum by documenting an adult case of heterotaxy and polysplenia who exhibited prototypical vascular anomalies, including an azygos continuation of the IVC, with no significant cardiac or biliary anomalies. This contrasts with the multiple pediatric polysplenia cases, which tend to exhibit complex congenital heart diseases, such as outflow tract obstructions and endocardial cushion defects, which present in up to 50-90% of pediatric patients [10]. A recent study found that only about 12% of patients with an interrupted IVC exhibited completely normal cardiac anatomy, showing how fortunate our patients' benign presentation is [11].

The constellation of CT findings in polysplenia syndrome is clearly distinctive when recognized with key features including multiple spleens, usually between two and six, in an abnormal location commonly located on the right side of the abdomen, with an absent normal splenic bed in the left upper quadrant [12]. The presentation of the liver may be midline or predominantly on the left side, as seen in our patients. The stomach's presentation can be ectopic, being more predominantly located on the right side. A hallmark finding seen in polysplenia syndrome is the interruption of the retrohepatic IVC with collateral venous return, as well as a typically dilated azygos, paralleling the aorta on the right, which drains the lower body venous blood to the superior vena cava [13]. Since the hepatic segments of the IVC are missing, the hepatic veins usually drain separately into the atrium [12]. These venous anomalies are usually present in the majority of polysplenia patients and can be easily identified on CT or MRI. Additional imaging findings can include bilateral bilobed lungs as well as a midline location of the abdominal organs. In our patient, the lungs demonstrated a mirror image of a bronchial pattern despite exhibiting left isomerism and abdominal features, further illustrating the complexity of heterotaxy. When multiple spleens are encountered on an imaging study, it should prompt a careful search for these associated anomalies in order to prevent misinterpretation of anatomy and guide appropriate follow-ups. 

The developmental distribution underlying situs ambiguous with polysplenia occurs early in embryogenesis, which establishes the left-right body axis symmetry. In early embryogenesis, normal asymmetric signal cascades genes like LEFTY and NODAL aid in ensuring the correct positioning of the organs. However, in polysplenia syndrome, there is believed to be a failure in the left-right pattern leading to a form of bilateral left-sidedness [11]. This left-sidedness leads to duplications of organs typically found on the embryological left side, such as polysplenia and bi-lobed lung structures, which become duplicated or mirrored. The development of the IVC involves a complex evolution of embryonic veins, involving the posterior cardinal, subcardinal, and supracardinal veins. The interruption of the hepatic segments of the IVC with azygos continuation that develops in this heterotaxy results from the failure of the right subcardinal vein to attach to the hepatic segments, leading to the persistent supracardinal pathway taking over the drainage [11]. This anomaly is strongly associated with left isomerism, which is different from asplenia, a right isomerism that often presents with a normally formed IVC and other distinct vascular anomalies, such as a left-sided SVC and a central liver [14]. The presence of multiple spleens in this heterotaxy arises from the duplication of splenic tissue due to the leftward lateralization signals occurring on both sides of the developing embryo [13]. The variations in embryological presentation can differ between individuals, explaining why our patient had normal cardiac development while other patients with the same syndrome may have severe heart malformation. 

Documentation of this heterotaxy anomaly in adult patients is crucial for clinical care. In our patient, specifically, the knowledge of her azygos continuation of the IVC is crucial if she ever requires certain procedures. For example, in the event of a deep vein thrombosis, placement of an IVC filter in the usual infrarenal location may be ineffective or impossible in our patient due to the interrupted IVC, requiring alternate strategies to be attempted. Similarly, in cardiopulmonary bypass or catheterization procedures that typically rely on cannulation of the IVC, careful planning must be conducted as the specialists must be aware that the venous return from the lower body is via the dilated azygos vein. There have been reported cases where an unrecognized interrupted IVC anatomy led to challenges during a cardiac catheterization and pacemaker placement, leading to complications until the anomaly was documented [12]. Furthermore, an interrupted IVC has been reported with an elevated risk of venous thrombosis in patients, inferred to be associated with the altered hemodynamics and collateral circulation. Another clinical consideration is the immunological capacity of the polysplenia, which can be associated with functional asplenia, or splenic tissue that is present but not fully effective. Although our patients with multiple spleens are presumably functional polysplenia patients may require an assessment of splenic function and appropriate vaccinations against encapsulated organisms as a precaution, similar to those of asplenic patients.

## Conclusions

The embryological disruption leading to situs ambiguous with polysplenia and azygos continuation of the IVC can be an incidental finding discovered in late adulthood with minimal symptoms. However, understanding these anatomical variations is necessary for more than academic education. These variations have real-life implications that span imaging interpretation and future surgical and interventional planning for these patients with altered anatomical positioning. Although this was an incidental finding, it may have large significant future relevance. Thus, a documentation of such vascular anomalies is crucial to mitigate future complications and to recognize early thoracoembolic events.
